# Understanding the Impacts of Molecular and Macromolecular Crowding Agents on Protein–Polymer Complex Coacervates

**DOI:** 10.1021/acs.biomac.3c00545

**Published:** 2023-10-10

**Authors:** Shanta Biswas, Alison L. Hecht, Sadie A. Noble, Qingqiu Huang, Richard E. Gillilan, Amy Y. Xu

**Affiliations:** † Department of Chemistry, Louisiana State University, Baton Rouge, Louisiana 70803, United States; ‡ Cornell High Energy Synchrotron Source (CHESS), Cornell University, Ithaca, New York 14853, United States

## Abstract

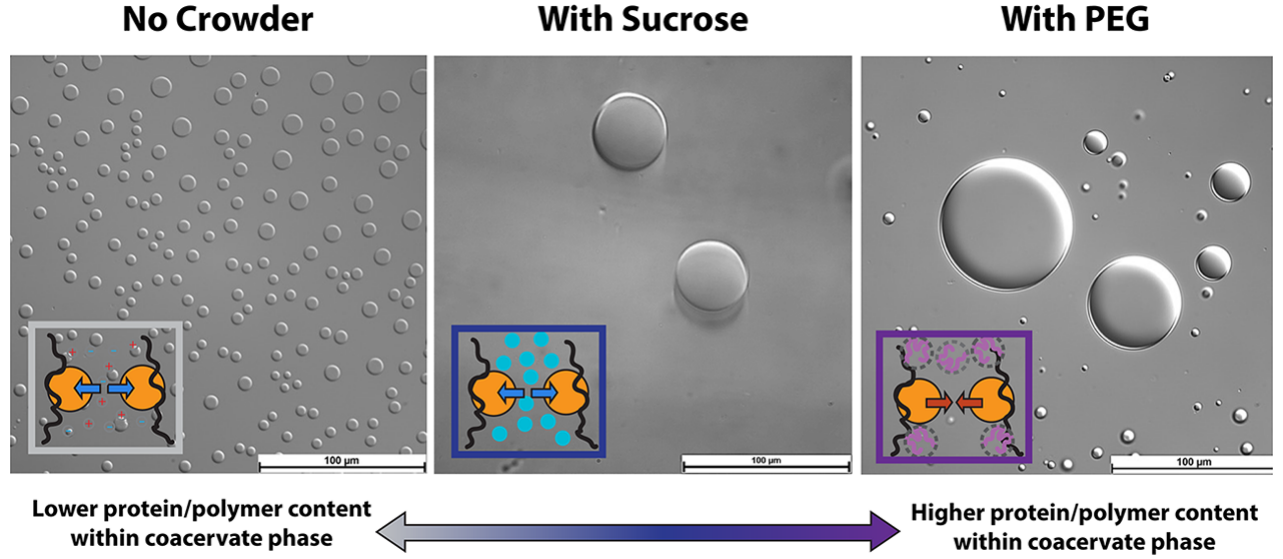

Complex coacervation refers to the liquid–liquid phase separation (LLPS) process occurring between charged macromolecules. The study of complex coacervation is of great interest due to its implications in the formation of membraneless organelles (MLOs) in living cells. However, the impacts of the crowded intracellular environment on the behavior and interactions of biomolecules involved in MLO formation are not fully understood. To address this knowledge gap, we investigated the effects of crowding on a model protein–polymer complex coacervate system. Specifically, we examined the influence of sucrose as a molecular crowder and polyethylene glycol (PEG) as a macromolecular crowder. Our results reveal that the presence of crowders led to the formation of larger coacervate droplets that remained stable over a 25-day period. While sucrose had a minimal effect on the physical properties of the coacervates, PEG led to the formation of coacervates with distinct characteristics, including higher density, increased protein and polymer content, and a more compact internal structure. These differences in coacervate properties can be attributed to the effects of crowders on individual macromolecules, such as the conformation of model polymers, and nonspecific interactions among model protein molecules. Moreover, our results show that sucrose and PEG have different partition behaviors: sucrose was present in both the coacervate and dilute phases, while PEG was observed to be excluded from the coacervate phase. Collectively, our findings provide insights into the understanding of crowding effects on complex coacervation, shedding light on the formation and properties of coacervates in the context of MLOs.

## Introduction

Complex coacervation is a type of liquid–liquid phase separation (LLPS) that occurs due to strong associative interactions between charged macromolecules. The resulting coacervate phase is enriched with interacting macromolecules and is in equilibrium with another liquid phase that is depleted in macromolecules. Complex coacervates can be prepared using a variety of macromolecules, including polysaccharides,^1−3^ proteins,^4−8^ nucleic acids,^9−13^ and synthetic polymers.^14−16^ Unique properties of complex coacervates, such as ultralow surface tension, high density, and tunable mechanical properties,^17−27^ enable their use in a wide range of applications. Moreover, adjustment of the interactions between macromolecules can modify the properties of coacervates. For example, changing the strength of electrostatic interactions between macromolecules can lead to changes in the composition and rheological properties of the coacervates.^17,18^

In addition to their use in food,^28−30^ pharmaceutical,^31−33^ personal care,^34−36^ and agriculture industries,^37^ the concepts of complex coacervation are being utilized in fundamental research to elucidate the formation of membraneless organelles (MLOs).^38,39^ The study of MLOs is an active area of research that offers a unique opportunity to investigate the fundamental principles underlying the phase behavior and self-assembly of biological macromolecules. MLOs often appear as dynamic microdroplets enriched with various proteins and nucleic acids that assist in hosting specific catalytic reactions or storing certain enzymes during stress.^40,41^ Numerous pairs of biomolecules have been utilized to form model complex coacervates that can serve as artificial MLOs to investigate their various features.^42−46^ While these model systems have been helpful in developing a fundamental understanding of the reversible compartmentalization behavior of biomolecules and their tendency to localize within a particular region,^47−49^ the majority of contemporary studies were conducted in diluted solutions. In living cells, MLOs are formed in a crowded environment with various molecules at high concentrations, accounting for 30–40% of the total cell volume.^50−52^ Therefore, it is essential to investigate model complex coacervate systems in an environment akin to the cytoplasm to gain a comprehensive understanding of MLO formation.

To recreate the crowded environment found within living cells, researchers have used crowding agents in many studies.^53−55^ These agents act as inert cosolutes, occupying significant volumes in the system without interacting with macromolecules that undergo LLPS to form MLOs. Crowding agents can be classified into two categories: molecular crowders and macromolecular crowders, based on their differences in molecule size, shape, flexibility, and occupied volume.^56,57^ Small molecules such as sucrose, trehalose, sorbitol, and glycerol have been studied as molecular crowders to improve the catalytic efficiency of enzymes and stabilize protein molecules against thermal stress.^58−60^ Macromolecules such as polyethylene glycol (PEG), ficoll, and dextran are also widely used to mimic the crowded environment within cells.^23,61−64^ The physical properties of molecular and macromolecular crowders differ, leading us to hypothesize that they could have different impacts on the behavior of charged macromolecules and thus the phase behavior of macromolecular complexes. Therefore, this study aims to investigate the effects of molecular and macromolecular crowding agents on the interaction mechanism as well as the mesoscale structure of a model protein–polymer complex coacervate system.

Previous research from our group demonstrated that bovine serum albumin (BSA) and poly(diallyldimethylammonium chloride) (PDADMAC) can form complex coacervate in a 50 mM NaCl solution at pH 7.^65^ Therefore, BSA and PDADMAC were chosen as a model protein–polymer coacervate system to examine the crowding effects. BSA is a globular protein with a molecular weight of 66.5 kDa and an isoelectric point of 4.7,^66^ which remains negatively charged at pH 7, whereas PDADMAC is a quenched polyelectrolyte that is positively charged in solution.^67^ To examine the effects of various crowding agents on the process of complex coacervation, sucrose and PEG were selected as representatives of molecular crowders and macromolecular crowders, respectively. The physical properties of prepared coacervates, such as the appearance and composition, were characterized using a wide range of microscopy and spectroscopy techniques. Moreover, small-angle X-ray scattering (SAXS) measurements were performed to characterize the effects of crowding on both the behavior and conformation of individual BSA and PDADMAC molecules as well as the internal structure of BSA/PDADMAC complex coacervates. Our study demonstrates that crowding agents, both molecular and macromolecular, led to the formation of larger coacervate droplets. A closer look at the crowding effects at the molecular level revealed that coacervates formed with sucrose had a structure similar to those formed without crowding agents, while coacervates formed with the presence of PEG were characterized by significantly more compact BSA/PDADMAC complexes with heavily overlapping BSA molecules. The difference in the internal structure of the coacervate formed with the presence of PEG could be explained by the effects of PEG on individual BSA and PDADMAC molecules. It was found that PEG led to an expanded conformation of PDADMAC and nondominating attractions among BSA molecules. These changes associated with individual macromolecules are expected to result in complex coacervates with distinct physical properties, including higher density and increased BSA incorporation. Overall, our study highlights the impacts of molecular and macromolecular crowding agents on the mesoscale structure and composition of protein–polymer complex coacervates, providing insights into their potential applications in various research fields.

## Experimental Section

### Materials

Bovine serum albumin (BSA) (CatNo. BP9706100) was purchased from Fisher Scientific, USA. Sucrose, PDADMAC (400–500 kDa, CatNo.409030), PEG (8 kDa, CatNo.P5413), and poly(4-styrenesulfonic acid) sodium salt (200 kDa, CatNo.561967) were purchased from Sigma-Aldrich, USA. Ultrapure water was used to prepare all solutions. All other reagents were of analytical grade and used without further purification.

### Preparation of BSA and PDADMAC Stock Solutions

In this study, BSA/PDADMAC complex coacervates were prepared under three different solution environments: 50 mM NaCl, 50 mM NaCl with 300 mM sucrose, and 50 mM NaCl with 3.30 mM PEG. These three solution environments are referred to as NaCl, sucrose, and PEG solutions, respectively, throughout the manuscript for clarity. The molar concentration of PEG was chosen to match that of sucrose, considering each ethylene glycol dimer as equivalent to the dimeric sucrose. To prepare the BSA stock solutions (approximately 100 mg/mL), an appropriate amount of BSA powder was added to each of the three different solutions, and the solution was allowed to solvate at 4 °C overnight to ensure complete hydration. PDADMAC stock solutions were prepared at a concentration of 2 mg/mL in each of the three different solutions.

### Preparation of BSA/PDADMAC Complex Coacervates

**Figure 1:**
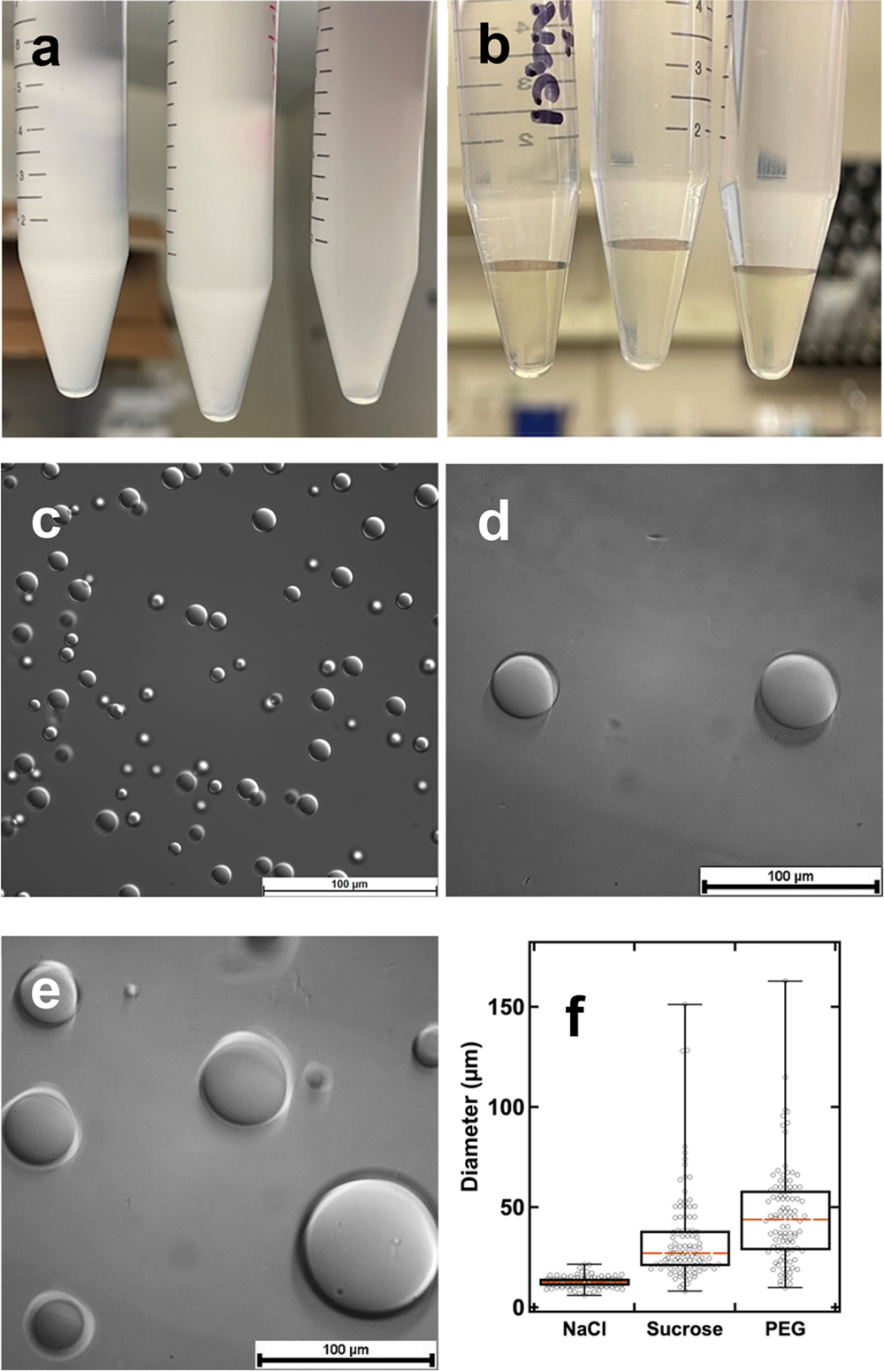
BSA/PDADMAC complex coacervate samples prepared in NaCl (left), sucrose (middle), and PEG (right) solutions before (a) and after (b) centrifugation. Microscopic images obtained from coacervate samples prepared in NaCl (c), sucrose (d), and PEG (e) solutions. Scale bars in these images represent 100 μm. (f) Box plot demonstrates the size distribution of coacervate droplets formed in three solution conditions. The lower and upper boundaries of the box represent the 25th and 75th percentile, respectively. The lower and upper limits represent the lowest and highest value, respectively. The median is represented by the red line lying in the middle of the box.

The BSA and PDADMAC stock solutions were combined to obtain a BSA to PDADMAC mass ratio (*r*) of 5, which corresponds to a charge ratio of ca. 7. Upon mixing, the solution immediately became turbid. The turbid solution was then centrifuged at 20 °C for 20 min at 4500 rpm, which resulted in the formation of two optically clear phases: the coacervate phase at the bottom and the dilute phase on top (as shown in Figure 1b). The samples were stored in the respective tubes in which they were initially prepared. The tubes were securely capped and sealed using parafilm, after which they were stored in a refrigerator at 4 °C.

### Viscosity Measurements

Viscosity values of NaCl, sucrose, and PEG solutions were measured using an m-VROC viscometer (Rheosense, USA) at 20 °C. Approximately 800 μL of each solution was loaded into a 1 mL glass syringe provided with the instrument. Multiple readings were performed at a flow rate of 800 μL/min followed by calculation of the average viscosity of the sample. The m-VROC viscometer cell chamber is equipped with an E02 chip, which is capable of measuring samples at a higher shear rate. Viscosity was measured as a function of the pressure drop as the fluid flew in the microfluidic channel. The slope calculated from the pressure drop was used to determine the viscosity, whereas the flow rate of the sample passing through the channel determined the apparent shear rate. The viscometer measures the apparent viscosity (η) of the sample,^68^ which can be calculated as
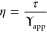
1where τ and ϒ_app_ are the shear stress and apparent shear rate, respectively. Here, ϒ_app_ is calculated from the slope obtained from the pressure versus sensor position graph recorded after each measurement. The equation to calculate τ can be presented as
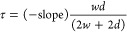
2where *w* and *d* are the width and depth of the E02 chip channel, which are 2 mm and 20 μm, respectively. The apparent shear rate, ϒ_app_, is calculated using the flow rate of the sample *Q* according to
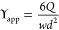
3

### Optical Microscopic Imaging

The coacervate droplets were observed by using a Leica DM6B microscope. To prepare microscopy slides, approximately 30 μL of the coacervate sample (the dense liquid phase shown in Figure 1b) was carefully collected from the bottom of the tube and placed in a concavity slide covered with a top cover slide. Optical images were taken in differential interference contrast (DIC) mode for better contrast. To determine the droplet size distribution, photos were taken at different spots for each sample, and the size of these droplets within these images were examined using ImageJ software.^69^ For each sample, at least 100 droplets of various sizes were captured and analyzed. The same set of samples was stored in a refrigerator at 4 °C for 25 days and then subjected to another round of microscopic examination.

### Protein Concentration Measurements

The concentration of BSA was determined by measuring the absorbance at 280 nm by a UV–vis spectrophotometer (UV 1600 PC Spectrophotometer, VWR, USA) with a molar extinction coefficient of 43,824 cm^–1^ M^–1^.^70^ To determine the concentration of BSA in the coacervate phase, the volume and BSA concentration in the supernatant was measured. The amount of BSA used to prepare the sample was known, and therefore, the amount of BSA within the coacervate phase was calculated by subtracting the remaining amount in the supernatant from the total mass of BSA used.

### Partition of PDADMAC in the Supernatant

To determine both the presence and relative quantity of PDADMAC remaining in the supernatant across three different crowding environments, we mixed the supernatant with a 10 mg/mL PSS solution. Since PSS is negatively charged, it strongly interacts with PDADMAC to form precipitates, resulting in a turbid solution. Control experiments were conducted by mixing PSS with BSA, PEG, and sucrose solutions, and none of these mixtures showed an increase in turbidity. Thus, it can be concluded that the increase in turbidity upon addition of PSS to the supernatant was solely attributable to the formation of precipitates between PSS and PDADMAC. As a result, the level of turbidity observed upon addition of PSS can be directly linked to the amount of PDADMAC present in the supernatant. The transmittance of these turbid samples was measured using a UV–vis spectrophotometer at a wavelength of 450 nm, and the turbidity of each sample was reported as (100 – % transmittance) as demonstrated in previous studies.^71,72^

### Measurement of Water Content within the Coacervate Phase

To measure water content in the coacervate phase, approximately 100 μL of the coacervate sample was loaded into a preweighed 1.5 mL ultracentrifuge tube. The tube containing the coacervate sample was weighed and then placed on a preheated heating block at 85 °C. After 4 h of drying, the tube was weighed again using the same analytical balance, and the percentage of water was calculated using eq 4.
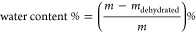
4where *m*_dehydrated_ is the mass of residue left after dehydration and *m* is the mass of coacervate taken to conduct the dehydration experiment.

### Size and Zeta Potential Measurements

The apparent sizes of BSA, PDADMAC, and their complexes when prepared in different solution environments were measured using a dynamic light scattering (DLS) instrument (Litesizer500, Anton Paar, USA) at 20 °C. The instrument was equipped with a single wavelength laser diode emitting a light of 658 nm. Prior to the measurements, all solutions were filtered through 0.45 μm syringe filters to remove impurities. Multiple measurements were performed for each sample to obtain an average hydrodynamic radius (*R*_h_). *R*_h_ was calculated using the Stokes–Einstein equation:
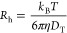
5where *k*_B_ is the Boltzmann constant, *T* is the absolute temperature, *D*_T_ is the translational diffusion coefficient, and η is solvent viscosity.

The zeta potential values (ζ) of BSA, PDADMAC, and their complexes when prepared in different crowding environments were also measured. Samples were loaded into a univette where the mobility of the particles was measured in the presence of an electrical field. The electrophoretic mobility (μ) was calculated (assuming the particles are spherical in shape) according to the following equation:^73^
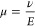
6where ν is the drift velocity of a dispersed particle (m/s) and *E* is the applied electric field strength. The results were reported as mean ζ potential of three readings, and standard deviations were calculated accordingly.

### Small-Angle X-ray Scattering (SAXS)

The SAXS measurements were conducted on the BioSAXS beamline (Sector 7A1) at Cornell High Energy Synchrotron Source (CHESS), located in Ithaca, New York, USA. Samples were loaded into quartz capillaries with an outer diameter of 1.5 mm, and the scattering patterns were recorded with an X-ray energy of 11.2577 keV. The scattering measurements covered the *q*-range from 0.0087 to 0.4 Å^–1^, which corresponds to a length scale from 2 to 72 nm. All SAXS profiles were reduced using the BioXTAS RAW software.^74^ Data analysis was performed using the NCNR analysis macro package built into IgorPro software.^75,76^ The double-logarithmic plot of *I*(*q*) vs *q* was obtained for each sample and used for data analysis. The scattering vector *q* is defined as
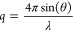
7where λ is the wavelength and 2θ is the scattering angle. For a system containing monodisperse, homogeneous, and isotropic dispersion of spherical particles, *I*(*q*) can be expressed as
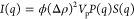
8where ϕ is the volume fraction of the particles, Δρ is the difference in scattering length density between the scattering particles and the solvent, *V*_p_ is the volume of the particle, *P*(*q*) is the form factor providing information on the size and shape of the scattering object, and *S*(*q*) is the structure factor that is related to the spatial arrangements of particles and thus contains information on the interparticle interactions.

In this study, the low-*q* region of the scattering profiles measured from complex coacervates were fitted using the polydisperse Gaussian coil model,^77^ whereas Kratky plots were generated to identify the correlation peak position in the intermediate-*q* region.^78^ SAXS measurements were also used to determine the effects of small and macromolecular crowders on protein–protein interactions (PPIs) among BSA molecules. As the BSA molecules were concentrated within the coacervate phase, PPIs among them became significant as BSA molecules came closer to each other at high protein concentrations. Understanding the effects of crowding on PPIs among concentrated BSA molecules will help explain the different physical properties of the coacervates formed with the presence of different crowding agents, as it is anticipated that the crowding agents can modulate the interactions between protein and polymer molecules and subsequently affect the complex coacervation process between them. Therefore, in this study, the structure factor of BSA was measured from a 100 mg/mL solution prepared in different crowding environments. Theoretical *S*(*q*) is calculated under the assumption that scattering objects are spherical and monodisperse. However, in many systems, the scattering objects may not be uniformly distributed and perfectly spherical, such as the BSA molecules in this study. Therefore, *S*(*q*) measured from scattering experiments were referred to as the effective structure factor *S*(*q*)_eff_.^79−81^ Experimentally, *S*(*q*) can be extracted from the scattering profiles by dividing out the contribution from *P*(*q*), as per eq 8. Therefore, the experimentally determined structure factor, *S*(*q*)_eff_, was obtained by dividing *I*(*q*) measured from the concentrated protein solution (100 mg/mL) by *I*(*q*) measured from the diluted solution (5 mg/mL), since it is anticipated that the structure factor is absent at such a dilute concentration. *S*(*q*)_eff_ can be represented as
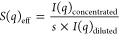
9where *I*(*q*)_diluted_ and *I*(*q*)_concentrated_ are the scattering profiles measured from diluted and concentrated protein solutions, respectively; *s* is the scaling factor for the given concentration where the scattering was measured. In this study, three different structure factor models were employed: (a) the hard sphere model, which assumes that steric repulsion is the only intermolecular interaction; (b) the Hayter–Penfold model, which includes additional Coulombic repulsions between molecules; (c) the Two–Yukawa model, which accounts for both attractive and repulsive interactions among the scattering objects.

## Results and Discussion

### Physical Properties of Molecular and Macromolecular Crowding Agents

Sucrose is a disaccharide composed of glucose and fructose with a molecular weight of 342 g/mol, whereas PEG used in this study is a hydrophilic polymer consisting of repeating −(CH_2_CH_2_O)– units and has an average molecular weight of 8000 g/mol. Sucrose and PEG have been considered as nonionic crowders which are unlikely to interact with BSA or PDADMAC via electrostatic interactions.^57,82^ DLS measurements were performed to measure *R*_h_ of sucrose and PEG molecules when they were dissolved in 50 mM NaCl solution. The *R*_h_ values of sucrose and PEG were 0.60 and 2.21 nm, respectively, consistent with previous studies.^83−86^ PEG exhibited a relatively small *R*_h_, suggesting that it adopted a random coil conformation in solution.^87,88^ The viscosity of the 50 mM NaCl solution was similar to that of pure water at approximately 1 cP. Adding 300 mM sucrose to the 50 mM NaCl solution led to a significant increase in viscosity to 1.33 cP. The viscosity of the 3.30 mM PEG solution was the highest at 1.89 cP. Parameters extracted during viscosity measurements are shown in Table S1. Collectively, sucrose and PEG demonstrate drastically different physical properties such as molecular weight, size, shape, and viscosity in aqueous solutions.

### Effects of Crowding Agents on the Size and Composition of BSA/PDADMAC Complex Coacervates

Upon mixing BSA and PDADMAC at a mass ratio of 5, instant turbidity was observed in all three solution environments, indicating the occurrence of phase separation (Figure 1a). After centrifugation, two optically clear liquid phases were observed (Figure 1b). The dense liquid phase at the bottom exhibited a faint yellow color, indicating the presence of highly concentrated BSA molecules; therefore, this dense liquid phase is the BSA/PDADMAC coacervate. The appearance of coacervates formed under different crowding conditions was observed using an optical microscope. The coacervate droplets were found to be the smallest in size when prepared in NaCl solution, with a median size of 12 μm and a narrow size distribution. However, with the addition of crowders, the size range of the coacervate droplets significantly broadened for both sucrose and PEG systems (Figure 1c–e). The median droplet size observed in the presence of sucrose was around 27 μm, while in the presence of PEG, the droplets were much larger with a median size of 46 μm (Figure 1e). To ensure that the size difference observed in different crowding environments was not due to time-dependent changes, three samples were subjected to microscopic examination after being stored at 4 °C for 25 days. The appearance and size of the coacervate droplets remained unchanged in all three crowding environments, indicating that the observed differences in size among the coacervate droplets were solely caused by the presence of crowding agents and that the coacervates remained stable over time (Figure S1).

**Figure 2:**
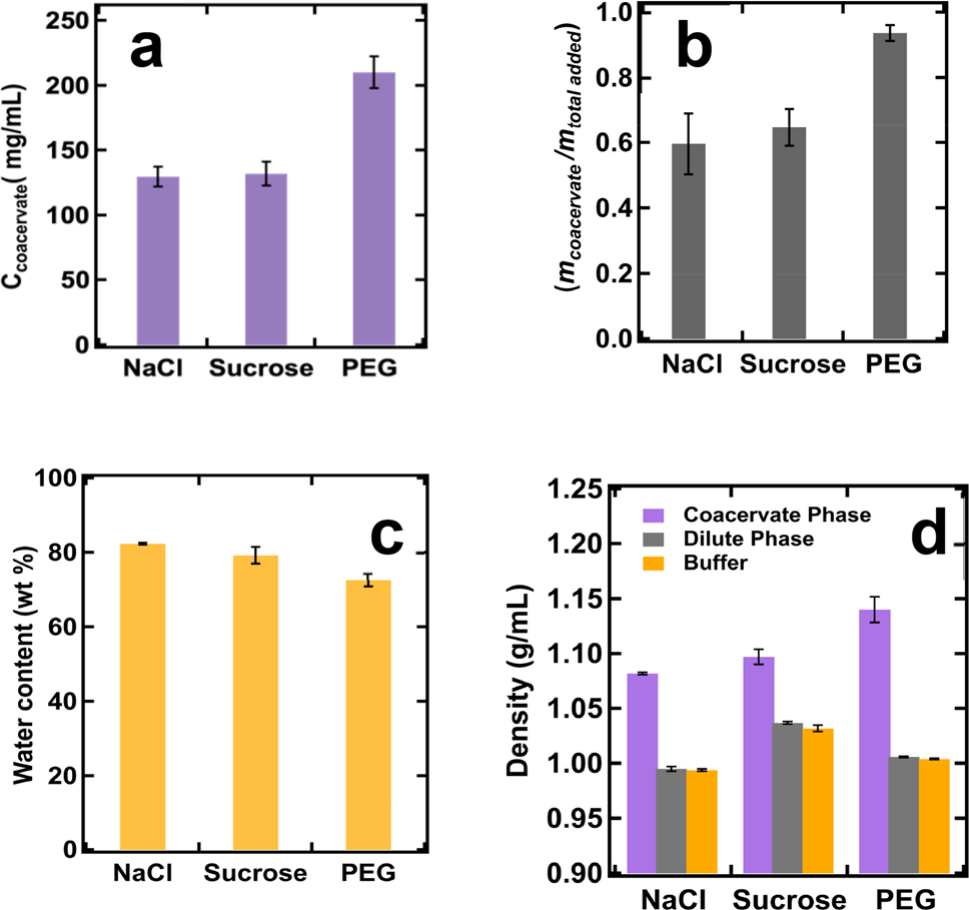
(a) BSA concentration within the coacervate phase (*C*_coacervate_). (b) The ratio of *m*_coacervate_/*m*_total_ of BSA. (c) Water content within the coacervate phase prepared in NaCl, sucrose, and PEG systems. (d) Density measured from the coacervate phase, dilute phase, and respective buffers under three solution conditions. Error bars correspond to one standard deviation from repeated measurements.

To better understand the impacts of molecular and macromolecular crowding agents on BSA/PDADMAC complex coacervates, we performed a series of experiments to determine the composition of coacervates formed in three different solution environments. First of all, we measured the BSA concentration in the coacervate phase (*C*_coacervate_). The *C*_coacervate_ value measured from the NaCl solution was 127 mg/mL, similar to that measured from the sucrose solution (Figure 2a). In contrast, the coacervate phase was much more concentrated with BSA (210 mg/mL) in the presence of PEG. We also compared the volume of coacervate formed in three different solution environments (Figure S2). The coacervate volume was slightly higher in sucrose solution than that in NaCl solution, whereas the volume of coacervate formed in PEG solution was slightly less than that measured from NaCl and sucrose solutions, despite the much higher BSA concentration measured within the coacervate phase. We compared the ratio between the amount of BSA in the coacervate phase (*m*_coacervate_) and the total amount of BSA added to the solution (*m*_total_) (Figure 2b). The *m*_coacervate_/*m*_total_ ratio represents the percentage of added BSA involved in the coacervation process. In a 50 mM NaCl solution, 58% of the total BSA was transferred into the coacervate phase. In the presence of sucrose, the amount of BSA involved in complex coacervation increased to 65%. Finally, when PEG was used as the crowding agent, almost all of the BSA molecules (approximately 94%) in solution were incorporated into the coacervate phase.

Unlike BSA, measuring the concentration of PDADMAC in the coacervate phase has been challenging. Nonetheless, we were able to determine the relative amount of PDADMAC remaining in the supernatant under three distinct solution conditions. For this purpose, we mixed the PSS solution with dilute phases collected from NaCl, sucrose, and PEG solution environments. A dilute phase containing PDADMAC will show turbidity upon mixing with a PSS solution due to the formation of PSS–PDADMAC precipitates. The turbidity of the three samples was recorded as 95%, 89%, and 10% for the dilute phases collected from NaCl, sucrose, and PEG solutions, respectively (Figure S3). These findings indicate that residual PDADMAC in the supernatant was most abundant in the NaCl solution and least abundant in the PEG solution. Therefore, it can be concluded that the coacervate phase contained the highest amount of PDADMAC when PEG was present, whereas substantially smaller amounts of PDADMAC contributed to the formation of coacervates in the NaCl and sucrose solutions. In addition to BSA and PDADMAC, the water contents within the three coacervate samples were also determined and compared. We found that the coacervate formed in the NaCl solution contained the highest amount of water at 82% (w/w). In the coacervate from the sucrose solution, water made up 78% (w/w) of the total mass. The coacervate formed in the PEG solution had the lowest water content at 72%(w/w) (Figure 2c).

Finally, we measured the densities of buffers, dilute phases, and coacervate phases in three different solution environments (Figure 2d). The results indicate that all coacervates had higher densities compared to their corresponding supernatants. However, the densities of the dilute phases in all conditions were similar to those of their respective buffers. Among the three environments, coacervates from the NaCl solution had the lowest density, and those from the sucrose solution showed a slight increase in density, while coacervates from the PEG solution had the highest density. Comparing the density difference between the coacervate and the dilute phases, it was found that the density difference was the smallest in NaCl solution, whereas the difference was more significant in the PEG solution. The greater density difference observed in the PEG solution is consistent with the observation that the coacervates formed in the presence of PEG sedimented the fastest among all three systems.

Collectively, we determined the composition of BSA/PDADMAC complex coacervates formed in three different crowding environments. Our results reveal that coacervate formed in NaCl solution (i.e., without crowding) contained the least amount of BSA and PDADMAC molecules but the largest amount of water. The density of the coacervate formed in the NaCl solution was also the lowest, aligning with its BSA, PDADMAC, and water content. The coacervate formed in the presence of the macromolecular crowder PEG contained the highest amounts of BSA and PDADMAC, as well as the least amount of water in the coacervate phase. The coacervate formed with sucrose exhibited a slight increase in BSA and PDADMAC content compared to the coacervate formed in the NaCl solution. However, this difference was not as significant as that observed in the coacervate formed in the presence of PEG. Overall, our results suggest that both molecular and macromolecular crowders can impact the size of the coacervate droplets. However, the composition of coacervates formed in the presence of PEG differs significantly from those observed from sucrose and NaCl solutions, indicating that PEG could exert different effects on BSA and PDADMAC molecules.

### Internal Structure of BSA/PDADMAC Complex Coacervates Probed by Small-Angle X-ray Scattering (SAXS)

**Figure 3:**
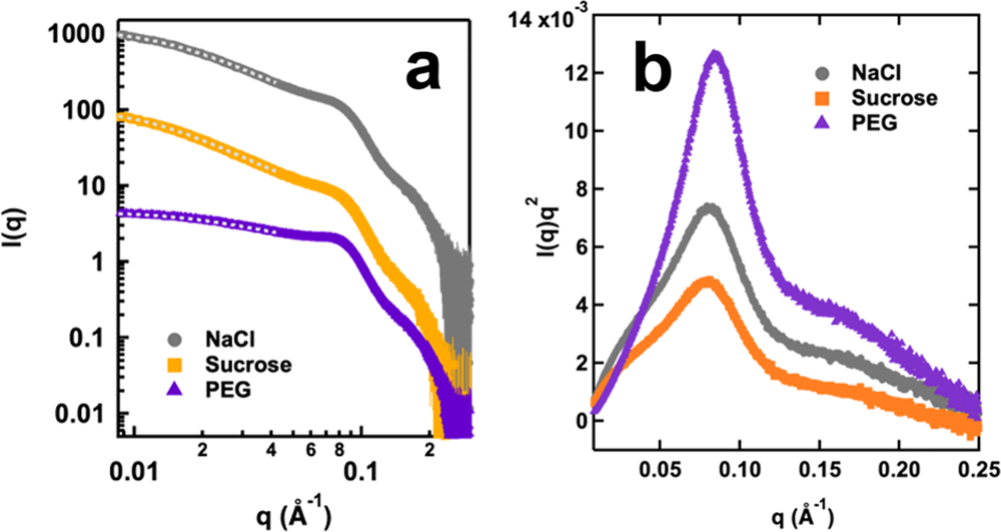
(a) SAXS profiles measured from BSA/PDADMAC coacervates formed in three different crowding environments. Gray dotted lines in the low-*q* region represent the Gaussian coil fit to the experimental data. Scattering profiles were offset to allow for better visualization. Error bars in scattering profiles were propagated from the relative uncertainties in the scattering intensity measurements based on counting statistics. (b) Kratky plots of SAXS profiles measured from coacervate samples under three solution conditions.

In this study, SAXS experiments were performed to investigate the assembly of BSA and PDADMAC molecules during LLPS to form complex coacervates. At pH 7, BSA is a negatively charged globular protein with a hydrodynamic radius around 3.8 nm,^89^ whereas PDADMAC is a positively charged polymer chain. It is anticipated that, upon electrostatic interactions, BSA molecules would interact with the charged backbone of PDADMAC chains to form BSA/PDADMAC intrapolymeric complexes. The scattering profiles measured from the three coacervate samples are shown in Figure 3a and are characterized by two features: an upturn in the low-*q* region (less than 0.04 Å^–1^) and a correlation peak in the intermediate-*q* region (at around 0.08 Å^–1^). In this study, BSA/PDADMAC complex coacervates were prepared at a mass ratio of 5, equivalent to a BSA to PDADMAC molar ratio of 34. Considering the possible conformation of BSA/PDADMAC complexes, we fit the low-*q* region using the Gaussian coil model, in which the BSA-bound PDADMAC chains (i.e., BSA/PDADMAC intrapolymeric complexes) were considered as the major structural elements within this length scale. From the Gaussian coil model, the radius of gyration (*R*_g_) of BSA/PDADMAC intrapolymeric complexes was obtained through model fitting. It was found that the average *R*_g_ of BSA/PDADMAC complexes was 10 nm when they were prepared in NaCl solution. The *R*_g_ value of the BSA/PDADMAC complexes increased slightly to 12 nm in sucrose solution. Compared to NaCl and sucrose solutions, the size of BSA/PDADMAC complexes formed in the presence of PEG reduced significantly to 6 nm, suggesting that the BSA/PDADMAC complexes had a more compact conformation.

In addition to the upturn observed at the low-*q* region, correlation peaks were observed in the intermediate-*q* region around 0.08 Å^–1^, a feature that is commonly observed from protein–polymer coacervate samples as well as concentrated protein solutions.^90−92^ A shared feature between coacervates and concentrated protein solutions is the significantly reduced distance between protein molecules, resulting in a more defined distance between adjacent protein molecules. Therefore, the close packing of BSA molecules within the coacervate samples likely contributes to the observed correlation peak at 0.08 Å^–1^. From the Kratky plot (Figure 3b), it can be seen that the correlation peak observed from coacervates prepared in NaCl and sucrose solutions were at the same *q*-value, both at 0.08 Å^–1^, corresponding to a repeating distance of approximately 7.9 nm, slightly larger than the reported hydrodynamic diameter of native BSA molecules at 7 nm.^93^ As previously discussed, this correlation peak likely arose from the close packing of BSA molecules within the coacervate phase. Considering the size of individual BSA molecules, it is reasonable to suggest that the peak position serves as an indicator of the center-to-center distance between adjacent BSA molecules, denoted as *d*_BSA_. With the addition of PEG, the correlation peak was shifted to a higher *q*-value at 0.085 Å^–1^, corresponding to a *d*_BSA_ value of 7.4 nm. The *d*_BSA_ value measured from the PEG system was considerably smaller than that measured from NaCl and sucrose solutions, indicating that the BSA molecules were more densely packed into the coacervate phase. Such a result is in line with the much higher BSA concentration measured from the coacervate prepared in PEG solutions (Figure 2a).

Therefore, SAXS measurements suggest that the internal structure of coacervates formed in NaCl and sucrose solutions was similar. The BSA/PDADMAC intrapolymeric complexes formed in NaCl and sucrose solutions exhibited random coil conformation with the BSA molecules decorated along the coiled PDADMAC chains. The distance between adjacent BSA molecules within the BSA/PDADMAC complexes was averaged to be around 7.9 nm. The internal structure of coacervates formed in the presence of PEG had a different structure, featuring more compact BSA/PDADMAC complexes with densely packed BSA molecules. This compact internal structure can be used to explain the significantly higher BSA concentration as well as the increased density observed in the coacervate phase in the presence of PEG. Although the further arrangement of BSA/PDADMAC complexes could not be characterized due to the limited *q*-range, the microstructure of the coacervates did appear to be influenced by the nature of the crowding agents.

### Effects of Crowding Agents on the Nonspecific Protein–Protein Interactions (PPIs) among BSA Molecules

**Figure 4:**
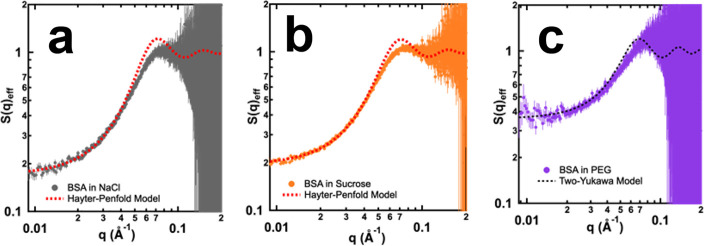
*S*(*q*)_eff_ profiles measured from 100 mg/mL BSA prepared in NaCl (a), sucrose (b), and PEG (c) solutions. Fits to the *S*(*q*)_eff_ profiles are represented by dotted curves. Error bars in scattering profiles were propagated from the relative uncertainties in the scattering intensity measurements based on counting statistics.

To better understand the effects of various crowding agents on BSA/PDADMAC complex coacervates, it is essential to examine the crowding effects on BSA and PDADMAC individually. Since BSA became more concentrated within the coacervate phase, SAXS measurements were performed to evaluate the nonspecific PPIs among concentrated BSA molecules in various solutions. The scattering profiles measured from dilute and concentrated BSA prepared in three different solution environments are presented in Figure S4. As depicted in Figure S4, the scattering profiles measured from 100 mg/mL BSA showed a decrease in *I*(*q*) toward the low-*q* region, indicating that the overall PPIs among BSA molecules were repulsive in nature.^94^ To better understand the various interactions contributing to the overall PPIs, the effective structure factor *S*(*q*)_eff_ was fitted using the appropriate models (Figure 4). The *S*(*q*)_eff_ profiles measured from 100 mg/mL BSA in NaCl and sucrose solutions were best fitted using the Hayter–Penfold model, which accounts for both volume exclusion and electrostatic repulsions. The *S*(*q*)_eff_ profile measured from BSA in the PEG solution was best fitted with the Two–Yukawa model that accounts for both repulsive and attractive interactions (details on *S*(*q*) model selection can be found in the Supporting Information).

**Figure 5:**
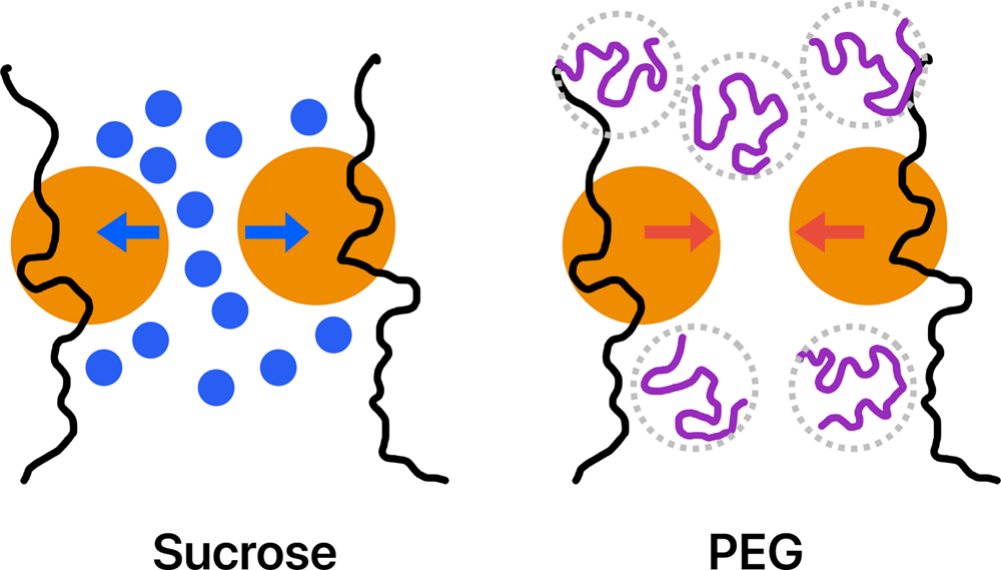
Schematic illustration of the effects of sucrose (blue spheres) and PEG (purple curves) on the interactions between two adjacent BSA molecules (orange spheres). The blue arrows represent the strong repulsions between BSA molecules due to charge and volume exclusion, whereas the red arrows represent the attractive depletion forces between adjacent BSA molecules due to the presence of PEG.

Fitting to the *S*(*q*)_eff_ profiles measured from three solution conditions indicated that molecular crowders, such as sucrose, did not change the nature of PPIs among the BSA molecules. In both NaCl and sucrose solutions, the overall PPIs among BSA molecules were dominated by repulsions arising from charge and volume exclusion. However, in the presence of macromolecular crowders such as PEG, although the overall PPIs were still dominated by repulsions, nondominating attractions were also observed among BSA molecules. The nondominant attractive forces observed among BSA molecules prepared in PEG solution can be attributed to the phenomenon of depletion forces.^95,96^ Due to the loss of configurational entropy in the vicinity of BSA molecules, PEG molecules were excluded from the surface of BSA, leading to the formation of a depletion layer around the protein. As the concentration of BSA increased, the depletion layers around adjacent BSA molecules started to overlap, effectively excluding PEG molecules from the intermediate space between the proteins. This resulted in an osmotic pressure imbalance with higher pressure outside the depletion layer than inside it. The net effect of this osmotic pressure imbalance was a weak attractive force that caused the BSA molecules to interact with each other.^96^ Different from PEG solution, where attractive forces were measured from concentrated BSA solutions, the interactions between BSA molecules were dominated by both volume exclusion and electrostatic repulsions in sucrose solution. Olsson et al. studied the roles played by trehalose and sucrose on the PPIs and found that sucrose can induce a well-defined protein–protein distance, thus separating the proteins and resulting in subsequent repulsive forces among protein molecules.^97^ Therefore, it can be seen that molecular and macromolecular crowders can lead to different effects on PPIs, which could be used to explain the drastically different composition and structure of complex coacervates formed in their presence (Figure 5).

In this study, we prepared BSA/PDADMAC complex coacervates using the same material and mass ratio but in three different crowding environments. We found that, in PEG solution, 94% of the added BSA molecules were incorporated into the coacervate phase, whereas only 65% of BSA underwent complex coacervation in the presence of sucrose. The larger amount of BSA within the coacervate phase formed in the presence of PEG can be attributed to the attractive forces among the BSA molecules. It is anticipated that BSA molecules first interacted with PDADMAC through electrostatic interactions due to the high availability of binding sites on the polymer backbone. The attachment of BSA onto the PDADMAC backbone led to an increased local concentration of BSA. At high BSA concentration, the protein molecules experienced both repulsive and attractive PPIs with the presence of PEG. The binding of BSA molecules to the PDADMAC chain resulted in a reduced protein charge, decreasing electrostatic repulsions between adjacent BSA molecules. As BSA molecules started to accumulate in the BSA/PDADMAC complexes, the attractive interactions among BSA (both polymer-bound and those in the vicinity of bound BSA molecules) increased, leading to further incorporation of BSA into the coacervate phase. The PDADMAC chains then hold BSA molecules in close proximity, leading to the formation of more compact BSA/PDADMAC network structures with the presence of PEG. In contrast, strong repulsive PPIs were measured from BSA molecules when prepared in NaCl and sucrose solutions. Therefore, when preparing complex coacervates with the same mass ratio, the amount of BSA incorporated into the coacervate phase was relatively similar in both conditions, as was the microstructure of the coacervates formed.

### Effects of Crowding on the Conformation of PDADMAC Molecules

**Figure 6:**
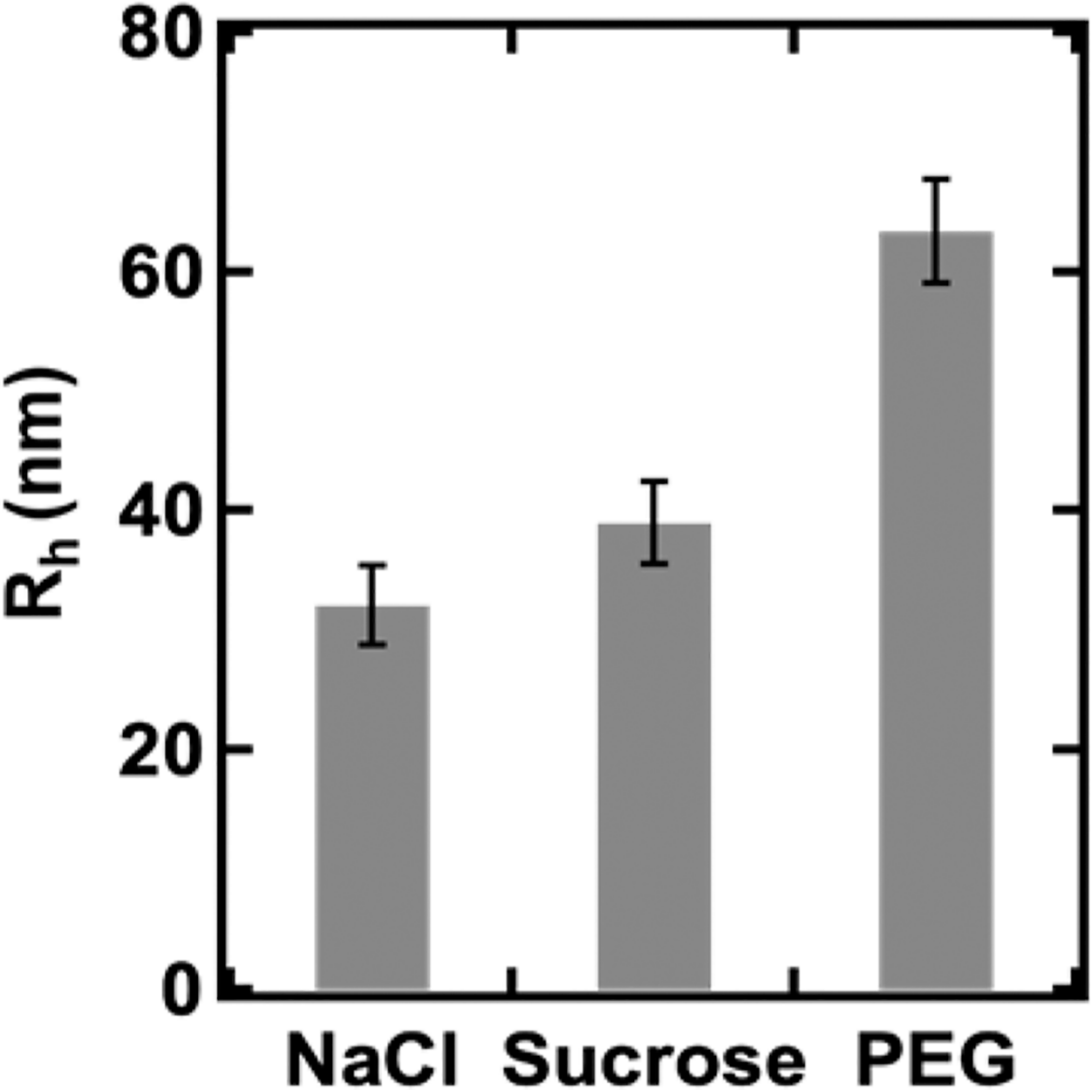
*R*_h_ values of PDADMAC were measured from different solutions. Error bars correspond to one standard deviation from the repeated measurements.

The conformation of PDADMAC was examined by using DLS in three different solution environments. The *R*_h_ value of PDADMAC showed a similar size in NaCl and sucrose solutions (∼30 nm), while a significant increase in size was observed in the presence of PEG (63 nm), suggesting a more expanded conformation was adopted by PDADMAC chains with the presence of PEG (Figure 6). It is anticipated that the expanded conformation of PDADMAC in the PEG solution could expose more charged sites that are accessible for BSA interactions. Therefore, in addition to the presence of attractive interactions between BSA molecules, this expanded conformation of PDADMAC could also contribute to the observed physical properties of complex coacervates formed with PEG, such as high protein content, increased density, and an enlarged droplet size.

### Molecular and Macromolecular Crowders Lead to Complex Coacervates with Various Microscopic and Macroscopic Properties

In this study, complex coacervates formed in three different solution environments were prepared and examined for their physical properties, including their overall appearance, composition, density, and internal structures. The presence of sucrose led to significantly larger coacervate droplets compared to the case in which no crowding was present, but the composition and internal structure of coacervates were not affected. In contrast, the presence of PEG resulted in significant changes in all aspects of coacervates, including much bigger droplet sizes, higher density, high BSA and PDADMAC contents, and the more compact internal structure featured with heavily overlapped BSA and PDADMAC molecules.

The differences in the physical properties of complex coacervates formed with molecular and macromolecular crowders are anticipated to be due to their effects on the individual protein and polymer molecules. It was found that the presence of PEG led to an expanded conformation of PDADMAC, as well as attractive depletion forces between BSA molecules. As a result, charged sites that were originally buried within the coiled conformation of PDADMAC became available for BSA to interact with, and the short-range attractions between BSA molecules could also promote more BSA incorporation into the coacervate phase. The attractive forces between BSA molecules can also explain the compact conformation of BSA/PDADMAC complexes: associative PDADMAC-bound BSA molecules could bring the expanded PDADMAC coils together, forming a collapsed chain conformation. In contrast, the conformation of PDADMAC and protein–protein interactions (PPIs) among BSA molecules was similar in both NaCl and sucrose solutions; as a result, the physical properties measured from BSA/PDADMAC complex coacervates appear to be similar when prepared without crowding and with sucrose as crowding agent.

### Molecular and Macromolecular Crowders Exhibit Different Partitioning Behaviors into the Coacervate and Dilute Phases

In addition to their impacts on the physical properties of complex coacervates, we are also interested in understanding the partitioning of crowders into the two liquid phases. As mentioned earlier, sucrose and PEG displayed distinct effects on the protein–protein interactions (PPIs) among BSA molecules with sucrose molecules positioned between adjacent BSA molecules, while PEG molecules were excluded from the vicinity of BSA molecules. Therefore, based on the different positioning of sucrose and PEG around BSA molecules, we hypothesized that they may exhibit different partitioning behaviors into the two liquid phases.

To validate our hypothesis, we first conducted mass spectroscopic measurements to determine the sucrose concentration in the BSA stock solution prepared with 300 mM sucrose as well as in the dilute phase after complex coacervation (i.e., before and after the addition of PDADMAC, respectively) (see the Supporting Information). The measured sucrose concentration in the dilute phase was found to be similar to that in the BSA stock solution, both around 300 mM (Table S2). This observation contradicts scenarios where sucrose would be excluded from the coacervate phase, as that would result in an increased concentration of sucrose in the dilute phase. Likewise, if sucrose were exclusively enriched in the coacervate phase, the sucrose concentration measured in the dilute phase would be close to zero. Consequently, based on the mass spectroscopy results, it can be inferred that sucrose molecules were distributed within both the coacervate and dilute phases. To further confirm the presence of sucrose in both phases, we performed FTIR analysis on the sucrose solution, supernatant, and coacervate collected from the sucrose environment. FTIR results show that sucrose molecules were present in both the coacervate and dilute phases (Figure S6).

To determine the partition of PEG into different liquid phases, we performed FTIR measurements on the PEG solution as well as the dilute and coacervate phases collected from the PEG solution (see the Supporting Information). The FTIR spectrum of the PEG solution demonstrates a characteristic peak at around 1100 cm^–1^, corresponding to the C–O–C stretching in the backbone of PEG^98^ (Figure S7). Such a characteristic peak was also evident in the dilute phase but disappeared in the FTIR spectrum collected from the coacervate, suggesting that PEG molecules were present in the supernatant but excluded from the dense coacervate phase. A previous study by Park et al. also demonstrates that PEG molecules do not partition into the coacervate phase but are solely distributed in the dilute phase.^99^

Therefore, our results suggest that sucrose molecules were present in both the dilute and coacervate phases, whereas PEG molecules were excluded from the coacervate phase and were present only in the dilute phase. Collectively, it can be seen that molecular and macromolecular crowders exhibit different partitioning behaviors in the coacervate and dilute phases. As previously hypothesized, sucrose and PEG are expected to have distinct effects on the PPIs among BSA molecules: sucrose molecules were distributed between BSA molecules, leading to separation among adjacent BSA molecules. Conversely, PEG molecules were depleted between adjacent BSA molecules, resulting in weak attractive forces between them. This discrepancy in partitioning behavior is in line with the hypothesized effects of sucrose and PEG on PPIs among BSA molecules and, consequently, their influence on the formation of BSA/PDADMAC complex coacervates.

## Conclusions

The effects of molecular and macromolecular crowding on a model protein–polymer complex coacervate system were investigated in this study. Our results reveal that sucrose, acting as a molecular crowder, had minimal impact on the physical properties of the coacervates. Conversely, PEG, serving as a macromolecular crowder, induced distinct physical properties in the coacervates, including higher density, increased protein and polymer contents, and a more compact internal structure. The differences observed between coacervates formed in sucrose and PEG solutions could be attributed to the effects of the crowders on the individual macromolecules, such as the conformation of PDADMAC and the interprotein interactions among BSA molecules. Moreover, our results show that sucrose was present in both the coacervate and dilute phases, while PEG was excluded from the coacervate phase. This discrepancy in partitioning behavior between sucrose and PEG aligns with the hypothesized effects of these crowders on modulating the interactions among BSA molecules, thereby influencing the formation of the BSA/PDADMAC complex coacervates. Therefore, understanding the underlying mechanisms of crowding effects on the individual macromolecules can provide valuable insights into the formation and properties of complex coacervates, which have significant implications for both fundamental research and practical applications.

## Supporting Information Available

Experimental parameters for viscosity measurements; microscopic images of complex coacervate samples, pre- and poststorage at 4 °C for 25 days; volume of complex coacervates formed under various solution conditions; characterization techniques for determining the presence of PDADMAC in the dilute phase; methodology for determining the density of coacervate samples; SAXS profiles from diluted and concentrated BSA samples and the criteria for selecting the appropriate structure factor model to fit *S*(*q*)_eff_ profiles; techniques employed to discern the partitioning of sucrose and PEG between the two liquid phases (PDF)
