# Supplementary material for: Understanding the Impacts of Molecular and Macromolecular Crowding Agents on Protein–Polymer Complex Coacervates
Source: Biomacromolecules. 2023 Oct 10;24(11):4771–82. doi: 10.1021/acs.biomac.3c00545 (PMC10646951; doi:10.1021/acs.biomac.3c00545)
Supplement: Supplementary file 1 — bm3c00545_si_001.pdf [file bm3c00545_si_001.pdf]

Understanding the Impacts of Molecular and Macromolecular Crowding Agents on Protein-Polymer Complex Coacervates

Shanta Biswas[1], Alison L. Hecht[1], Sadie A. Noble[1], Qingqiu Huang[2], Richard E. Gillilan[2], Amy Y. Xu[1]*

[1]Department of Chemistry, Louisiana State University, Baton Rouge, Louisiana, 70803, United States.

[2]Cornell High Energy Synchrotron Source (CHESS), Cornell University, Ithaca, New York 14853, United States.

Viscosity Measurements

Table S1. Parameters extracted from viscosity measurements for NaCl, Sucrose, and PEG solutions at 20 °C using m-VROC™ viscometer. Three consecutive measurements were performed for each sample.

Sample	Υ_{app} (s^{-1})	τ (Pa)	Apparent viscosity, η (mPa•s)	Flow rate, Q (µL/min)	Calculated average viscosity from apparent viscosities (mPa•s)
NaCl	97952.5	97.5	0.995	799.3	0.997
		98.1	1.002	799.3	
		97.3	0.994	799.3	
Sucrose	97952.5	131.6	1.343	799.5	1.334
		127.9	1.306	799.3	
		132.5	1.353	799.5	
PEG	97952.5	184.5	1.883	799.3	1.889
		185.2	1.891	799.3	
		185.5	1.894	799.3	

Appearance of coacervate droplets before and after 25 days

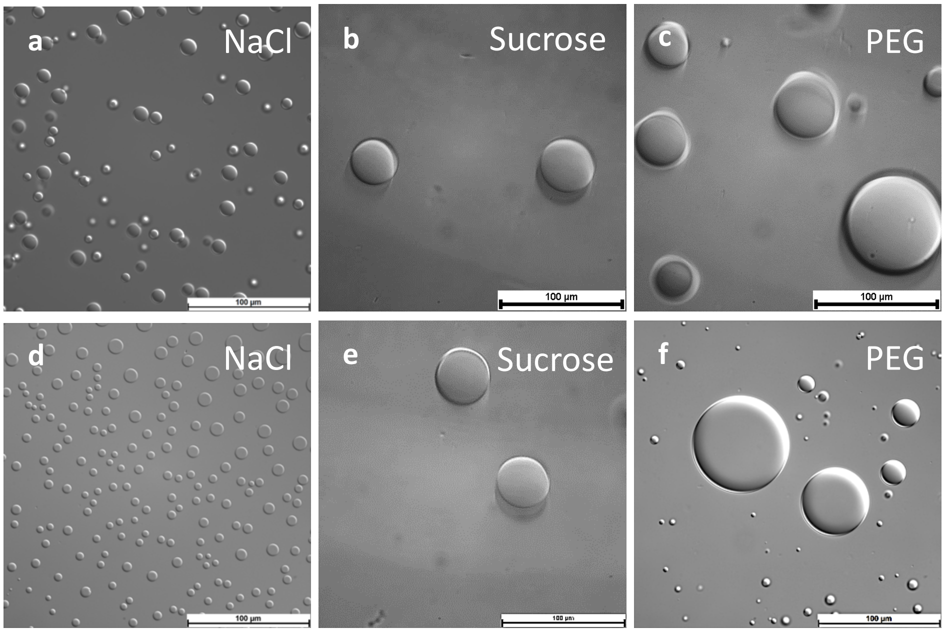

Figure S1. Microscopic images obtained from freshly prepared BSA/PDADMAC complex coacervates (a-c) and those after storing at 4 °C for 25 days (d-e) in three different solution environments.

Physical Properties of Complex Coacervates Formed in Different Crowding Environments

Volume of BSA/PDADMAC complex coacervates formed in three different crowding environments were measured and represented below in Figure S2.

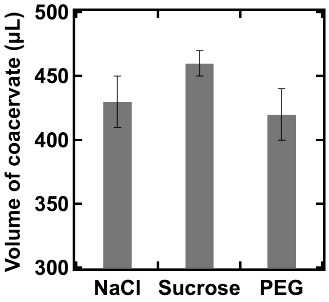

Figure S2. Volume of the coacervate phase ($V_{coacervate}$) in three solution conditions. Error bars correspond to one standard deviation from repeated measurements.

Elucidating the Presence of PDADMAC in the Dilute Phase

To determine the presence of residual PDADMAC in the dilute phase, we added 10 mg/mL solution of Poly(4-styrenesulfonic acid, sodium salt) (PSS) with a molecular weight of 200,000 g/mol to supernatants from different crowding systems. Given that PSS is negatively charged, it is expected to interact strongly with the positively charged PDADMAC, forming precipitates. Immediate turbidity was observed upon the addition of PSS to PDADMAC solution, confirming the formation of PSS/PDADMAC precipitates (Figure S3a). As a control, we also added PSS to BSA solutions to verify that PSS does not cause BSA aggregation (Figure S3a). Thus, it can be concluded that if turbidity arises upon the addition of PSS to the dilute phase, it is solely due to the formation of PSS/PDADMAC complexes. This turbidity can then be used as an indicator to determine the relative amount of PDADMAC present in the dilute phases of NaCl, sucrose, and PEG solutions. As shown in Figure S3b and S3c, the addition of PSS to the dilute phase resulted in significant turbidity in the NaCl and sucrose systems, indicating a large amount of residual PDADMAC in the dilute phase. In contrast, the PEG sample remained almost transparent, suggesting a minimal amount of PDADMAC in the dilute phase. Overall, these findings imply that almost all PDADMAC is sequestered within the coacervate phase in the PEG system, while significant amounts of PDADMAC still remain in the dilute phase for the NaCl and sucrose systems.

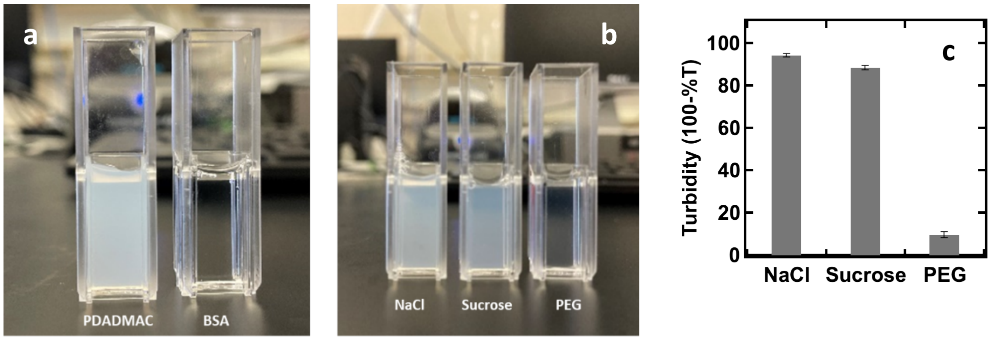

Figure S3. (a) Appearance of PDADMAC (left) and BSA (right) solutions upon addition of PSS. (b) Appearance of dilute phases collected from NaCl (left), sucrose (middle) and PEG (right) systems upon addition of PSS. Turbidity of these samples indicates the presence of PDADMAC. (c) Turbidity results measured from different supernatant samples after addition of PSS. The greater the turbidity of the sample, the more PDADMAC is present in the supernatant. Error bars correspond to one standard deviation from repeated measurements.

Measurement of Coacervate Density

To measure the density of complex coacervate sample, a 1.5 mL ultracentrifuge tube and a 200 µL pipette tip were weighed together by an analytical balance (W_1). The same pipette tip was then used to aspirate 100 µL of coacervate sample, and then the pipette tip containing 100 µL of coacervate sample was transferred back to the same ultracentrifuge tube. The ultracentrifuge tube containing the pipette tip and 100 µL coacervate sample was weighed again on the same balance (W_2). Therefore, the weight of 100 µL coacervate can be calculated as (W_2-W_1), and subsequently, the density of coacervate can be calculated using the volume of coacervate (which is 100 µL in this case). Multiple measurements were performed for each sample.

SAXS Profiles of Diluted and Concentrated BSA Solutions

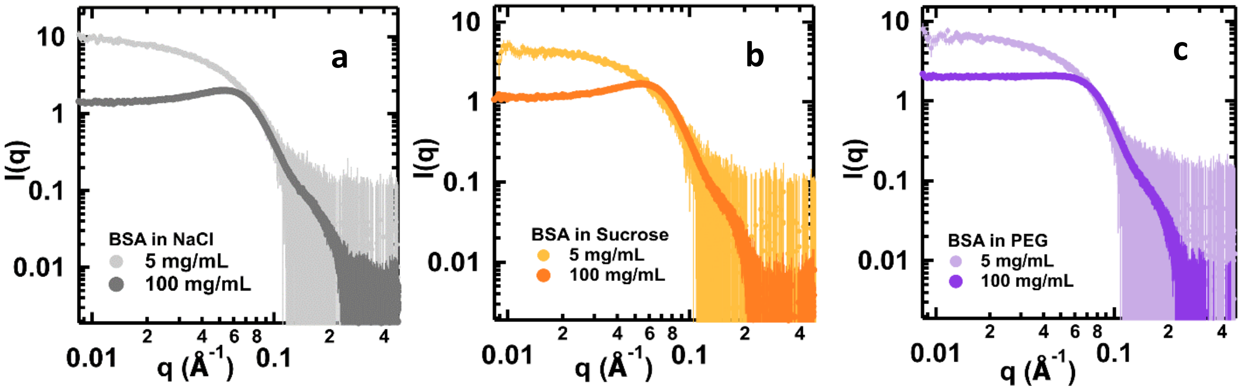

Figure S4. SAXS profiles measured from diluted (5 mg/mL) and concentrated (100 mg/mL) BSA samples prepared in (a) NaCl, (b) sucrose, and (c) PEG buffer conditions.

Selection of Structure Factor Models to Fit $S(q)_{eff}$ profiles

In this study, the effective structure factor profiles $S(q)_{eff}$ obtained from concentrated BSA prepared in various solution environments were fitted using appropriate models to account for various repulsive and attractive interactions present among individual protein molecules[1, 2]. Three models were used to fit $S(q)_{eff}$ profiles obtained from different samples these include: (1) the hard sphere model, where the steric repulsion is considered to be the only intermolecular interaction; (2) the Hayter–Penfold model, where additional Coulomb repulsions between molecules are also considered; and (3) the Two–Yukawa model, where both attractive and repulsive interactions are taken into account[2-6]. To determine which model is most appropriate, we compare the $S(q)$ profiles derived from the hard sphere model to the experimental $S(q)_{eff}$ profiles, as illustrated in Figure S5. In the case of NaCl solution, the $S(q)$ profile derived from the hard sphere model lies above the experimental $S(q)_{eff}$ profile, indicating the presence of additional repulsive interactions between protein molecules. Therefore, the Hayter-Penfold model, which includes charge repulsion, is used to fit the $S(q)_{eff}$ profile measured from NaCl solution. Similarly, the $S(q)$ profile derived from hard sphere model is higher than the $S(q)_{eff}$ profile measured from sucrose solution, suggesting that additional repulsive forces are at play. In contrast, for PEG solution, the $S(q)$ profile derived from hard sphere model lies below the $S(q)_{eff}$ profile, implying the presence of attractive interactions between protein molecules. In this instance, the Two-Yukawa model, which accounts for additional attractive interactions was employed to fit the $S(q)_{eff}$ profile. Therefore, we performed comparisons between experimental $S(q)_{eff}$ profile and theoretical $S(q)$ profile derived from the hard sphere model to discern the presence of additional repulsive or attractive forces beyond volume exclusion effects. It is also worth noting that the fit and the experimental data do not align well in the high-q region.

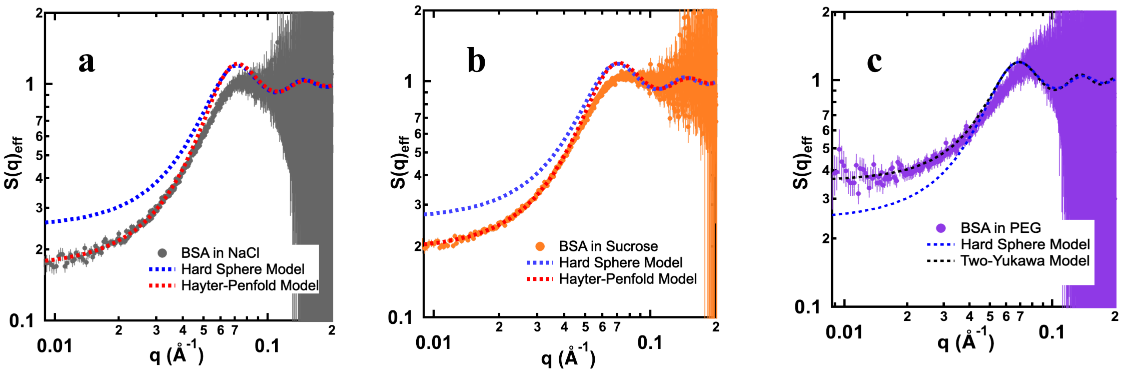

Figure S5. Summary of $S(q)_{eff}$ profiles measured from BSA prepared in (a) NaCl, (b) sucrose, and (c) PEG solution environments. In all figures, *S(q)* profile derived from hard sphere model is shown and represented as blue dotted curves. Compare the positioning between the $S(q)_{eff}$ and *S(q)* derived from the hard sphere model, either Hayter-Penfold model (in case of NaCl and sucrose) or Two-Yukawa model (in case of PEG) was chosen to fit the $S(q)_{eff}$ profile.

Determining the Distribution of Sucrose into the Coacervate and Dilute Phases

To examine the presence of sucrose in both liquid phases, we performed two types of experiments. First of all, we used mass spectroscopy to measure sucrose concentration in the BSA stock solution prepared with 300 mM sucrose, as well as in the dilute phase after complex coacervation (i.e., before and after the addition of PDADMAC, respectively). To do this, a stock solution of 300 mM sucrose in 50 mM NaCl was prepared. From this stock solution, diluted standard solutions were prepared for calibration, and the method was validated using the respective mass spectrometry instrument (Bruker amazon speed ETD). The calibration plot was constructed using concentrations ranging from 1.5 µM to 30 µM, demonstrating high linearity and sensitivity. To ensure the sucrose concentration in both the BSA stock solution and the dilute phase fell within the range of concentrations in the calibration plot, appropriate dilutions were made. The obtained sucrose concentrations for both samples were determined using mass spectrometry and are presented in Table S2. Mass spectroscopy results shown in Table S2 imply that sucrose could present in the coacervate phase due to the similar amount of sucrose concentration measured from both BSA solution and dilute phase.

Table S2. Sucrose concentration determined by mass spectroscopic measurements

Sample	Measured conc. from Mass Spec. (µM)	Concentration after counting for dilution factor (µM)	Conc. (mM)
BSA Solution (before adding PDADMAC)	29.95	299,500	299.5
Dilute Phase (after adding PDADMAC)	29.83	298,300	298.3

To further confirm the presence of sucrose in both phases, we performed FTIR analysis on the sucrose solution, supernatant and coacervate collected from the sucrose environment. To this end, FTIR spectra of the various samples were recorded using an Alpha-II compact FT-IR spectrometer (Bruker, Germany). Prior to every measurement the sample stage was cleaned with 70% ethanol to eliminate any possible contamination. About 20 µL of each sample was placed directly on to the sample stage. Each spectrum was collected by cumulating 32 background and 32 sample scans with a resolution of 4 cm^{-1} and a wavenumber range of 4000 - 400 cm^{-1}. Two distinct peaks were found to present in all three samples: one at 1055 and the other at 1001 cm^{-1}, attributing to the C-O-C stretching vibrations in the sucrose molecule[7](Figure S6). Such results imply that sucrose molecules were present in all three samples, confirming that sucrose is partitioned in both coacervate and supernatant phases.

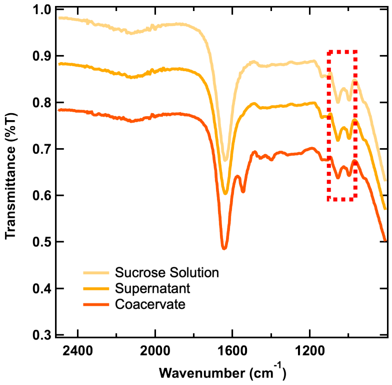

Figure S6. FTIR spectra collected from sucrose solution, supernatant and coacervate phase prepared in sucrose solution. Spectra are offset along the Y-axis for better visualization. Peaks attributing to the C-O-C stretching vibrations are highlighted in the dotted red box.

Determining the Distribution of PEG into the Coacervate and Dilute Phases

To examine the presence of PEG in different phases, FTIR measurements were also performed. Similarly, approximately 20 µL of various samples was placed directly on to the sample stage, including the PEG solution, supernatant and coacervate prepared in PEG solution environment. Each spectrum was collected by cumulating 32 background and 32 sample scans with a resolution of 4 cm^{-1} and a wavenumber range of 4000 - 400 cm^{-1}. In the FTIR spectrum collected from PEG solution, a characteristic peak was observed at around 1100 cm^{-1}, corresponding to the C-O-C stretching in the backbone of PEG (Figure S7). In particular, the peak at 1100 cm^{-1} is present in the PEG solution, the dilute phase, but disappeared from the coacervate phase. Such results suggest that PEG, as a macromolecular crowder, is excluded from the dense coacervate phase.

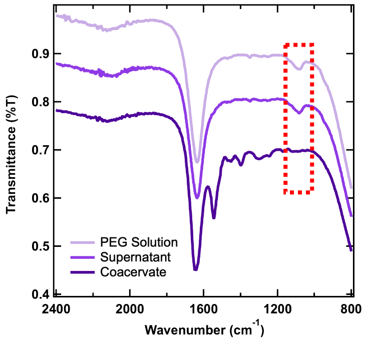

Figure S7. FTIR spectra measured from PEG solution, dilute phase and coacervate phase in PEG system. Spectra are offset along the Y-axis for better visualization. Characteristic peak attributed to the C-O-C stretching at around 1100 cm^{-1} is highlighted in the red box.

References

1. Xu, A. Y.; Clark, N. J.; Pollastrini, J.; Espinoza, M.; Kim, H. J.; Kanapuram, S.; Kerwin, B.; Treuheit, M. J.; Krueger, S.; McAuley, A.; Curtis, J. E., Effects of Monovalent Salt on Protein-Protein Interactions of Dilute and Concentrated Monoclonal Antibody Formulations. *Antibodies (Basel)* **2022,** *11* (2), 24.
2. Castellanos, M. M.; Clark, N. J.; Watson, M. C.; Krueger, S.; McAuley, A.; Curtis, J. E., Role of Molecular Flexibility and Colloidal Descriptions of Proteins in Crowded Environments from Small-Angle Scattering. *J. Phys. Chem. B* **2016,** *120* (49), 12511-12518.
3. Xu, A. Y.; Castellanos, M. M.; Mattison, K.; Krueger, S.; Curtis, J. E., Studying Excipient Modulated Physical Stability and Viscosity of Monoclonal Antibody Formulations Using Small-Angle Scattering. *Mol. Pharm.* **2019,** *16* (10), 4319-4338.

4. Chen, S. H.; Broccio, M.; Liu, Y.; Fratini, E.; Baglioni, P., The two-Yukawa model and its applications: the cases of charged proteins and copolymer micellar solutions. *J. Appl. Crystallogr.* **2007,** *40*, S321-S326.
5. Kaieda, S.; Lund, M.; Plivelic, T. S.; Halle, B., Weak Self-Interactions of Globular Proteins Studied by Small-Angle X-ray Scattering and Structure-Based Modeling. *J. Phys. Chem. B* **2014,** *118* (34), 10111-10119.
6. Hayter, J. B.; Penfold, J., An Analytic Structure Factor for Macroion Solutions. *Mol. Phys.* **1981,** *42* (1), 109-118.
7. Feng, M.; Hu, X.; Yin, Y.; Liang, Y.; Niu, J.; Yao, J., Structural analysis of oxidized sucrose and its application as a crease-resistant crosslinking agent. *Polymers* **2022,** *14* (14), 2842.
